# Supplementary material for: Persistence to Medications for Benign Prostatic Hyperplasia/Benign Prostatic Obstruction-Associated Lower Urinary Tract Symptoms in the ASL TO4 Regione Piemonte (Italy)
Source: Healthcare (Basel). 2022 Dec 17;10(12):2567. doi: 10.3390/healthcare10122567 (PMC9778582; doi:10.3390/healthcare10122567)
Supplement: Supplementary file 1 [file healthcare-10-02567-s001.zip › healthcare-1970025-supplementary.pdf]

Supplementary Materials

Persistence to Medications for Benign Prostatic Hyperplasia/Benign Prostatic Obstruction-Associated Lower Urinary Tract Symptoms in the ASL TO4 Regione Piemonte (Italy)

Lucrezia Greta Armando [1], Raffaella Baroetto Parisi [2], Elisa Remani [2], Mariangela Esiliato [2], Cristina Rolando [2], Valeria Vinciguerra [2], Abdoulaye Diarassouba [2], Clara Cena [1] and Gianluca Miglio [1,3,*]

[1] Department of Drug Science and Technology, University of Turin, Via Pietro Giuria 9, 10125 Turin, Italy
[2] Struttura Complessa Farmacia Territoriale ASL TO4, Regione Piemonte, Via Po 11, 10034 Chivasso (TO), Italy

[3] Competence Centre for Scientific Computing, University of Turin, Corso Svizzera 185, 10149 Turin, Italy
* Correspondence: gianluca.miglio@unito.it; Tel.: +39-0116707150

Drug prescription networks

A network approach was adopted as a data mining technique to investigate the complexity of drug prescriptions (Miglio et al., 2021). In particular, data on prescriptions of each $ATC_{i,L}$ (where i refers to drug classification at the given ATC level L) and prescriptions of pairs $ATC_{i,L}$–$ATC_{j,L}$ (with $i \neq j$) were considered. In a drug prescription network, the nodes were the $ATC_{i,L}$s found in the dataset and represented the number of patients prescribed with drug i within ATC-level L during the follow-up. The edges were the pairs $ATC_{i,L}$–$ATC_{j,L}$s and represented the number of patients prescribed with the drug pairs i and j within ATC-level L during the follow-up.

All analyses were performed using the R statistical and programming language (version 4.2.0; https://cran.r-project.org/) and the add-on package igraph.

Miglio, G.; Basso, L.; Armando, L.G.; Traina, S.; Benetti, E.; Diarassouba, A.; Baroetto Parisi, R.; Esiliato, M.; Rolando, C.; Remani, E.; Cena, C. A network approach for the study of drug prescriptions: analysis of administrative records from a local health unit (ASL TO4, Regione Piemonte, Italy). *Int J Environ Res Public Health* **2021**, 18(9), 4859.

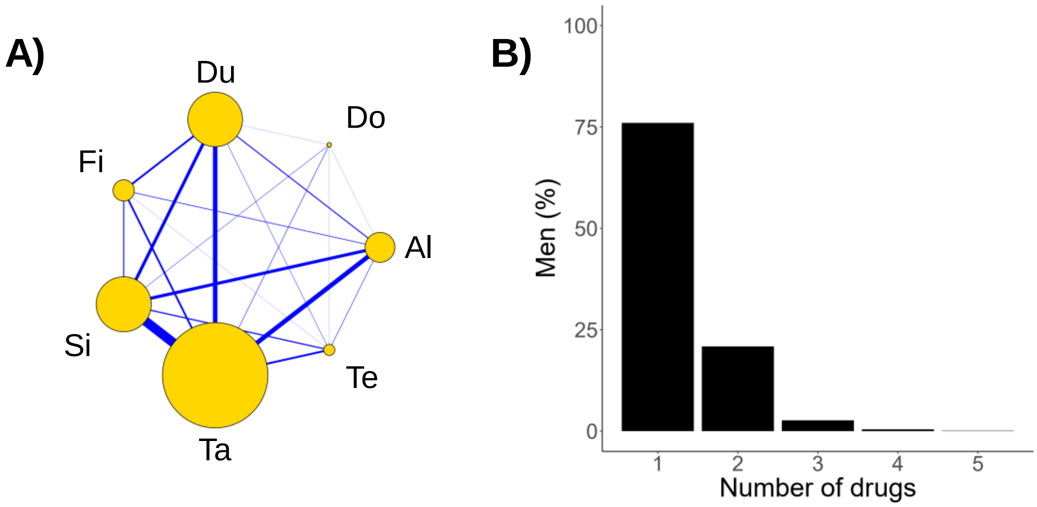

Figure S1. Prescription of drugs for the treatment of BPH/BPO-associated LUTS in the overall study population. (**A**) Data on drug prescriptions were analysed to generate a drug prescription network that correspond to the level 5 of the ATC classification system. Nodes represent the seven study drugs: alfuzosin (Al), doxazosin (Do), dutasteride (Du), finasteride (Fi), silodosin (Si), tamsulosin (Ta) and terazosin (Te). Node diameter and edge thickness were proportional to the number of patients prescribed with the individual ATC_is and the pairs ATC_i–ATC_j within the ATC level 5, respectively. (**B**) Distribution of patients with respect to the number of drugs prescribed.

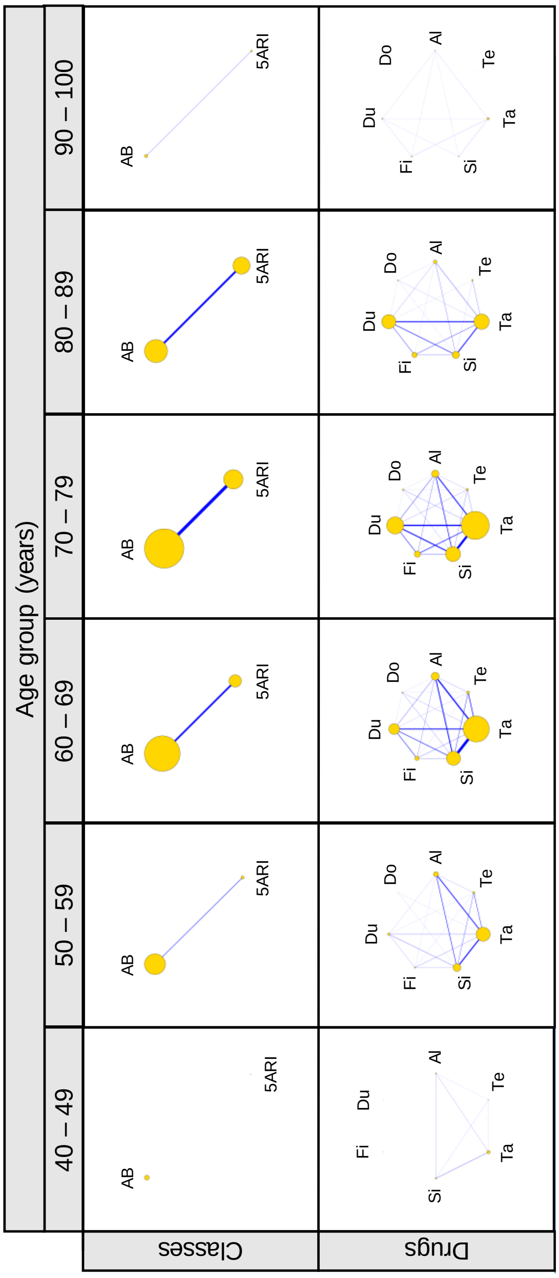

Figure S2. Drug prescription networks in the pre-specified age groups. Data on drug prescriptions were analysed to generate a drug prescription network that correspond to either the level 4 (pharmacological classes) or the level 5 (drugs) of the ATC classification system. Nodes represent either the two pharmacological classes: AB and 5ARI; or the seven study drugs: alfuzosin (Al), doxazosin (Do), dutasteride (Du), finasteride (Fi), silodosin (Si), tamsulosin (Ta), and terazosin (Te). Node diameter and edge thickness were proportional to the number of patients prescribed with the individual ATC_is and the pairs ATC_i–ATC_j within the ATC level 5, respectively.
